# The effects of breakfast on short-term cognitive function among Chinese white-collar workers: protocol for a three-phase crossover study

**DOI:** 10.1186/s12889-017-4017-1

**Published:** 2017-01-18

**Authors:** Zhenchuang Tang, Na Zhang, Ailing Liu, Dechun Luan, Yong Zhao, Chao Song, Guansheng Ma

**Affiliations:** 1 National Institute for Nutrition and Health, Chinese Center for Disease Control and Prevention, Xi Cheng District Beijing, 100050 People’s Republic of China; 2 Liaoning Center for Disease Control and Prevention, He Ping District Shenyang, 110058 People’s Republic of China; 3 School of Public Health and Management, Chongqing Medical University, Yu Zhong District Chongqing, 400016 People’s Republic of China; 4 Department of Nutrition and Food Hygiene, School of Public Health, Peking University, 38 Xue Yuan Road, Hai Dian District Beijing, 100191 People’s Republic of China

## Abstract

**Background:**

As the first meal of the day, breakfast plays an important role in supplying energy and nutrients, which are critical to working and learning activities. A three-phase crossover study was designed to investigate the effects of breakfast on cognitive function among Chinese white-collar workers. The planned study protocol is presented.

**Methods:**

A total of 264 participants aged 25–45 years will be recruited from Shenyang and Chongqing. Self-administered questionnaires will be used to collect information on age, gender, marital status, education level, occupation, smoking habits, drinking habits, and breakfast behaviours. The participants will be randomly assigned to 3 equal-sized groups (Groups A, B, and C) and will be provided with a nutrition-adequate breakfast, a nutrition-inadequate breakfast, or no breakfast, respectively. Each participant will receive the breakfast treatment on the basis of assignment to one of three sequences (ABC/BCA/CAB). Each participant will complete a battery of cognitive tests assessing short-term memory, attention, and working memory 120 minutes after breakfast. Mood will be measured through a self-administered questionnaire assessing the dimensions of positive and negative frames of mind. Additionally, fasting blood glucose and postprandial 2-hour blood glucose levels will be tested with a blood-glucose meter (Roche ACCU-CHEK®-Performa). All the participants will take all the tests in three successive weeks, and the order of presentation will be counter-balanced across groups.

**Discussion:**

The present study will be the first investigation of the effect of breakfast food type and quality on cognitive function amongst white-collar workers in China. We predict that a nutrition-adequate breakfast, compared with a nutrition-inadequate breakfast and no breakfast, will significantly improve short-term cognitive function. The results of this study should provide scientific evidence of the effect of breakfast quality on cognitive function and provide scientific data to inform nutrition education strategies and promote a healthy lifestyle.

**Trial registration:**

Chinese clinical trial registry (Primary registry in the WHO registry network) Registration number: ChiCTR-IPR-15007114. Date of registration: August 25, 2015.

## Background

As the first meal of the day, breakfast plays an important role in supplying energy and nutrients [1]. After sleeping through the night, the human body needs to refresh and regain energy and nutrients in time to engage in physiological activities. A high-quality breakfast can provide adequate energy and nutrients for working and learning activities [2]. In contrast, a low-quality breakfast leads to lower work efficiency, a large compensatory increase in food intake at the next meal and excessive burden on the gastrointestinal tract [3]. Previous studies have indicated that skipping breakfast no or consuming a low-quality breakfast is harmful for health [4, 5]. An adequate breakfast can not only maintain the body’s needs for activities but also reduce the risk of diabetes, osteoporosis, obesity, cardiovascular and cerebrovascular disease and other chronic diseases [6]. Research findings have demonstrated that people who eat breakfast every day have a 35 to 50% lower risk of obesity and diabetes than their counterparts who do not eat breakfast or eat breakfast occasionally [7].

Another study has revealed that skipping breakfast or eating a low-quality breakfast have a negative effect on cognitive function [8], thus resulting in a decline in brain excitability, the emergence of a slow response and a reduction in attention. In the morning, glycogen stores are significantly depleted after digestion over the course of the night [9]. Glucose is the main fuel for brain function, and optimal cognitive function requires the maintenance of a stable blood glucose level [10]. Breakfast has a direct effect on blood glucose levels and, in turn, blood glucose levels have a direct effect on cognitive function [11, 12]. In general, the brain performs best when the blood glucose level is in the range of 80–120 mg/dL [13]. With the gradual depletion of blood glucose and, consequently, energy consumption, people begin to feel hunger and fatigue and experience a decline in cognitive function [14]. A number of studies have reported that skipping breakfast lowers cognitive function and work efficiency [15–17]. Therefore, breakfast is considered to be the most important meal of the day for nutritional intake as well as work performance.

Along with the advanced socio-economic development in China, the nutritional status of residents has improved. However, owing to the lack of nutritional knowledge and awareness, the importance of breakfast has not been paid sufficient attention. In one study, 19.6% of participants reported rarely eating breakfast [5]. The percentage of students in primary school, junior high school and high school who eat breakfast every day has been reported to be 97.0, 92.4 and 93.4%, respectively; however, the nutritional quality of their breakfast was found to be generally poor [18].

To date, studies on breakfast and its effects on cognitive function have been conducted mainly on primary and secondary school students in China [16, 19]. Few studies on adults have been reported. Thus, to understand the effects of breakfast on the short-term cognitive function of adults, a three-phase crossover study was designed and will be conducted with white-collar workers in two cities in China. The aim of this study is to investigate whether breakfast consumption has an effect on adults’ short-term cognitive function. The effects of three types of breakfast differing in type of quality and energy on short-term cognitive function, blood glucose level, hunger, satiety and mood in adult white-collar workers will be studied.

## Methods/Design

### Study hypothesis

Participants who eat a nutrition-adequate breakfast will have better cognitive performance than those who eat a nutrition-inadequate breakfast or do not eat breakfast.

### Sample size calculation and participants

In a related study, the average cognitive performance value of people who ate a nutrition-adequate breakfast, a nutrition-inadequate breakfast and no breakfast has been found to be 36.6, 25.9 and 26.8, respectively [20]. Using these results as reference values, in this study, power is set at 0.8, SAS programming was used, and the necessary sample size was calculated. According to the SAS procedures described by Hu [21], we calculated the sample size as 117 in one city. If the dropout rate is 10%, a total of 130 participants will be needed. Given that the participants will be divided into three groups, and to achieve equal group sizes, 44 participants will be needed for each group. Therefore, 132 participants will be needed for the three groups to include an equal number of men and women in one city. Because this study will be conducted in two cities, 264 total participants will be recruited.

### Participants and study design

Participants will be recruited from two cities, Shenyang and Chongqing, China. Shenyang is located in northeast China, whereas Chongqing is located in southwest China.

The inclusion criteria will be: white-collar workers aged 25–45 years involved mainly in office work, who engage in brain work and are amenable to skipping breakfast.

The exclusion criteria will be: individuals with mental illness, oral disease or other severe diseases; pregnant women and nursing mothers; individuals who have a tendency to lose weight; and individuals who never eat breakfast.

**Fig. 1:**
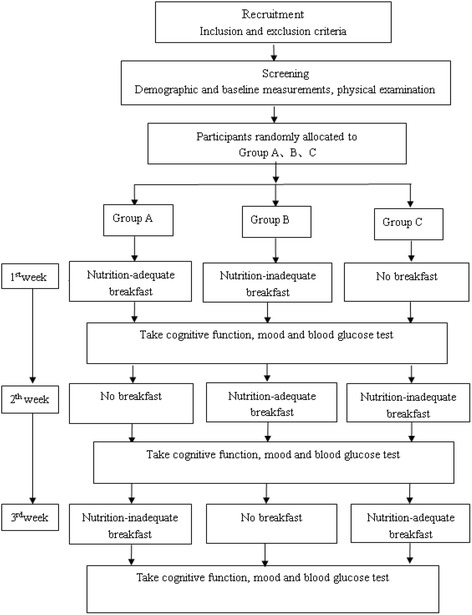
The study design

This crossover study will last for three weeks. Participants will be randomly assigned to one of three groups (Group A, Group B, and Group C). In the first week, Group A will be provided with a nutrition-adequate breakfast, Group B will be provided with a nutrition-inadequate breakfast, and Group C will be provided with no breakfast. Each participant will complete tests of blood glucose level, mood, hunger and satiety, and cognitive function. In the second week, Group A will be provided with no breakfast, Group B will be provided with a nutrition-adequate breakfast, and Group C will be provided with a nutrition-inadequate breakfast. Each participant will complete blood glucose level, mood, hunger and satiety, and cognitive function tests. In the third week, Group A will be provided with a nutrition-inadequate breakfast, Group B will be provided with no breakfast, and Group C will be provided with a nutrition-adequate breakfast. The blood glucose level, mood, hunger and satiety, and cognitive function will be tested again. All participants will take the tests in three successive weeks, and each participant will be tested in three breakfast treatments: nutrition-adequate breakfast, nutrition-inadequate breakfast, or no breakfast. The study design is presented in Fig. 1.

### Screening questionnaires

A screening questionnaire will be used to acquire general information about participants and to exclude participants for medical reasons (including anaemia or other blood disorders, food allergies, diabetes or glucose intolerance, other acute or chronic illnesses/diseases, colour blindness, severe learning disabilities and mood disorders). The screening questionnaire is a combination of self-designed questionnaire items and a standardized questionnaire. The self-designed questionnaire includes items to measure demographic characteristics, including age, gender, marital status, education level, occupation, smoking and drinking habits and frequency of breakfast eating. The standardized questionnaire includes a breakfast nutrition survey and a breakfast and nutrition knowledge survey.

### Breakfast treatment

According to the Chinese Dietary Guidelines [22] and Chinese Dietary Reference Intakes [23] as well as the food taboos and preferences of the participants [24], three types of breakfast will be prepared: a nutrition-adequate breakfast, a nutrition-inadequate breakfast, and no breakfast. The energy and nutrients of the nutrition-adequate breakfast and the nutrition-inadequate breakfast are shown in Table 1.

**Table 1:** Energy and nutrients from the nutrition-adequate breakfast and nutrition-inadequate breakfast

	Nutrition-adequate breakfast		Nutrition-inadequate breakfast	
	Male	Female	Male	Female
Food				
Congee (g)	200	200	200	200
Steamed bread (g)	120	80	60	30
Bean curd (g)	40	30	0	0
Eggs (g)	50	50	50	50
Beef (g)	40	30	0	0
Vegetables (g)	120	120	120	120
Fruits (g)	80	80	0	0
Macronutrient				
Protein (g)	17.9	12.9	8.3	9.3
Fat (g)	10.9	10.6	6.7	6.7
Carbohydrates (g)	126.4	90.4	87.1	73.5
Energy (kcal)	670	540	330	270

The nutrition-adequate breakfast will be composed of grains, animal protein (meat, eggs), soy products, vegetables and fruits, and it will provide energy to meet the requirements of 18- to 49-year-old adults who engage in light physical activity. The nutrition-adequate breakfast will provide 675 kcal (2250 kcal × 30%) for males and 540 kcal (1800 kcal × 30%) for females.

The nutrition-inadequate breakfast will provide half the energy of the nutrition adequate breakfast. The energy from the nutrition-inadequate breakfast will be 330 kcal for males and 270 kcal for females.

Participants in the no breakfast treatment will not eat breakfast. Therefore, they will ingest 0 kcal at breakfast.

### Cognitive function tests

The cognitive function tests will consist of short-term memory, learning and memory, and attention tests, which have been used to detect differences in cognitive function induced by glucose administration in previous studies [8, 20, 25]. Cognitive function tests will be administered in the same order to each participant for the three weekly testing times: (1) Mood, (2) Hunger and satiety, (3) Short term memory, (4) Learning and memory, and (5) Attention.

It is anticipated that the test duration time will be approximately 30 minutes.

#### Mood

The self-reported mood questionnaire that will be used was developed from the Positive Affect and Negative Affect Scale (PANAS) [26]. It was modified from previous research for Chinese adults. Twenty words will be used to assess mood [27]. All responses on a 5-point scale ranging from “not at all” to “extremely” will be analysed.

#### Hunger and satiety

Hunger and satiety data will be obtained to determine whether the observed effects are associated with changes in hunger or satiety. Sense of hunger and satiety will be assessed by using Stunkard’s method [28]. Standardized instructions and paper visual analogue scales (0–100 mm) will be used. A visual analogue scale will be provided that will display a construct so that its extreme left edge will represent the absence of the characteristic (e.g., no hunger at all or no feeling of fullness). The extreme right side of the construct will represent the highest possible degree of the characteristic, which, in this particular case, will be the greatest intensity of hunger imaginable. If satiety is being measured, this edge will indicate complete or maximum satiety. Participants will be instructed to place a vertical line through the horizontal axis, with the distance from the left edge corresponding to the current degree of hunger and satiety on the provided scale that encompasses a spectrum ranging from zero (not present) to the maximum (highest level), from the left edge to the right edge.

#### Short-term memory

A “digit span” task will be used to assess short-term memory. The participants will be instructed to listen and repeat what they hear. For example, if the investigator says “1 2 3”, the participant will repeat “1 2 3”. Words will be presented at a rate of one word per second. After a correct repetition by the participant, the next set of numbers will be increased by one. If the participant does not repeat the numbers correctly, the investigator will provide another set of words of the same length to the participant. Two incorrect repetitions will result in the termination of the test. Each participant will receive both a forward and a backward version of this task. The backward version will require the subject to repeat the numbers in reverse order. Thus, in continuing with the above example, the participant will repeat “3 2 1”. A total score (forward recall + backward recall) will then be used to assess overall paragraph recall performance.

#### Learning and memory

Learning and memory will be assessed by using the digit-symbol coding method, which mainly measures digital decoding ability, visual memory, visual attention and operating speed. A continuous performance task will be used to evaluate attention, through focusing on the vigilance component of attention. This task’s evaluation will consist of providing, for example, nine digit-symbol pairs (e.g., 1/一, 2/丄, 3/], 4/□, 5/∪, 6/○, 7/Λ,8/X,9/=) followed by a list of digits. Under each digit, the participant will be instructed to write down the corresponding symbol as quickly as possible. The number of correct symbols within the allotted time will be measured (90 seconds). The test results will provide an indicator of speed during involvement with activities such as visual search, attention, mental flexibility, and motor function.

#### Attention

A table composed of eight letters (A, B, C, E, H, K, N, X) will be provided, and participants will be asked to remove a specified letter under specific conditions. For example, participants will be asked to delete B, which follows H (with the suppression condition of Aventura Karimov lettering). In the provided tables, each letter will appear 150 times, and 1200 letters will be provided. The test time will be 2 minutes, and each test will use the same alphabet. Before performing the test, the investigator will explain the test’s rules. The reading velocity, mistake rate, index of mental capacity (IMC) as measured by the Number Cancellation Test and test time, and correct number of tracking lines as measured by the Visual Tracking Test will be calculated.

#### Creativity

Four types of description words, such as “usually red”, “usually round”, “usually noisy” and “usually with wheels”, will be provided. Within 5 minutes, participants will be asked to list as many different items as possible that fit the above descriptions. For example, the description “usually blue” can be expected to be associated with the sea, a fountain pen, the colour of water, or blue jeans, all of which are commonly associated with the colour “blue”.

#### Language logic

Language logic involves a series of logical presentations of right and wrong. For example, “a is in front of b” means that a and b or ab are the correct sequences. In such cases, the answer is right. However, in this context, the statement that “b is not behind a” means that an answer of “a and b” in this case would be scored as wrong, as the answer is erroneous.

The logical test results (i.e., the completed and the correct number of responses) will be evaluated. They will be defined as:

$$ \operatorname{Completed} \operatorname {number} = \operatorname{number} \operatorname {of} \operatorname {completed} \operatorname {questions} \operatorname {in}\ 3 \operatorname {minutes} $$

$$ \operatorname{Correct} \operatorname {number} = \operatorname{the} \operatorname {correct} \operatorname {number}/\operatorname{the} \operatorname {completed} \operatorname {number} $$

### Study procedure

**Fig. 2:**
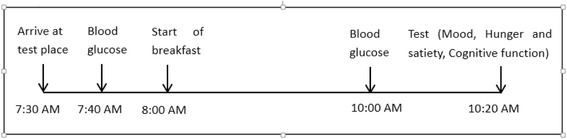
The study process

Each participant from each of the three groups will receive the designated breakfast and complete the tests for three successive weeks, and the order of presentation will be counter-balanced across the groups. All participants will be instructed to fast from 20:00 pm the night before the study. The next morning, the participants will arrive at the designed location at 07:30 am. All the participants will rest in a seated position for 10 minutes and provide a finger-prick blood sample for the blood glucose test (blood glucose meter, Roche ACCU-CHEK®-Performa). Subsequently, participants from the groups that consume a nutrition-adequate and a nutrition-inadequate breakfast will have 15 minutes to ingest their breakfast in the designed location under the supervision of investigators until the breakfast is completely eaten. The breakfast will be delivered by the investigators. During this interval, the participants in the no breakfast treatment group will rest for 15 minutes. After 120 minutes, a second finger-prick blood sample from each participant for the blood glucose test will be collected. Then, all the participants will complete a series of tests on mood, sense of hunger and satiety, and cognitive function. All the tests will be performed under similar laboratory conditions (quiet room, 22–24 °C). Testing will not occur on Mondays, because the diets of the participants over the weekend is likely to be more varied than usual and, in the case of poorly nourished participants, lower in energy intake. The study procedure is shown in Fig. 2.

### Quality control

A study manual will be provided before the survey, and one training will be conducted for all the investigators in the two cities. With the appropriate implementation and application of the unified method, a standardized face-to-face questionnaire will be administered.

Standardized experimental equipment and measurement methods will be used in the two cities to enable easy data interoperability. In addition, to ensure the same composition and quality of the breakfasts, the two cities will use the same breakfast recipes.

The data will be collected and entered by trained staff members from the Liaoning Center for Disease Control and Prevention and Chongqing Medical University. Double entry will be performed, and the data will be compared to identify and correct any discrepancies.

### Data management and entry

All the questionnaires will be collected, stored in a single file and regularly checked and rechecked. The questionnaires will be encoded and entered in a timely manner with the use of the Epidata 3.1 double entry test results.

### Analysis strategy

Statistical analyses will be conducted using SAS 9.2 (SAS Institute Inc., Cary, NC). Descriptive statistics will be calculated for all the variables under examination. Anthropometric measurements at baseline will be analysed to examine the influence of the factors on breakfast behaviour by using multivariate regression analysis, with age, sex, income and education level included in the model. Repeated-measures analysis of variance (ANOVA) will be used to examine cognitive function performance, with breakfast condition (Group A, Group B, or Group C) as the within-subjects variable and sex as the between-subjects variable. Significant confounding variables (in addition to gender), including satiety, mood score before the cognitive function tests, and time between breakfast and the first cognitive function test, will all be included initially as covariates in the two-way ANOVA. The significance level will be set at <0.05. The Bonferroni adjustment method will be used to perform the pairwise comparisons across different breakfast types. The significance test level will be ɑ' = 0.01667 (0.05/3). A modified t-test for gender differences will be used, and the significance level will be set at <0.05. All *P*-values will be two-tailed.

## Discussion

According to the design of the experiment, all the participants will receive three breakfast treatments in different stages to determine whether differences in cognitive function exist among the different groups. This experimental design will ensure that there is a balance in the confounding variables in each group and will eliminate the bias caused by participants’ characteristics. In addition, to ensure consistency in the composition and quality of the breakfast conditions, the same breakfast recipes will be used in the two cities, and one breakfast company will be responsible for the production and the distribution of the different breakfasts. In addition, breakfast samples will be sent to the Chongqing Food and Drug Inspection Institute for the measurement of energy and composition.

One challenge that we will face in this study will be the dropout rate and participant compliance. Participants will need to be present for three consecutive weeks. Some participants might fail to complete the entire study because of business travel, tardiness, resignation and other reasons. We will fully explain the principles and requirements to the participants before the study begins to ensure compliance.

The present study will have both strengths and weaknesses. This will be the first investigation of the associations among breakfast quality and energy and cognitive function among adult white-collar workers in China. The sample size will provide sufficient power to detect relatively small effects. It is expected that a high-quality breakfast will improve short-term cognitive function, whereas a low-quality breakfast will reduce short-term cognitive function. Furthermore, baseline information on the general demographic characteristics (including age, gender, marital status, education level, occupation, smoking habits, and drinking habits), frequency of breakfast eating, and body composition will be collected. More than 260 blood samples from participants will be tested, thus providing sufficient power to explore the effects of blood glucose on cognitive function. In addition, satiety and mood will be measured to address some of the potential confounding variables in this study. One limiting factor is the type of cities that were selected in this study, because China is very large. Additionally, there are various types of breakfasts with diverse nutritional values. In this study, we will restrict our analysis initially to Chongqing and Shenyang only, noting that imposing certain constraints to represent them as being nationally representative would be restrictive.

In summary, “breakfast”, which is derived from the combination of “break” and “fast”, is primarily consumed after a restful night of voluntary starvation. Individuals wake up requiring immediate nourishment and “break” their “fast” by consuming the first and most important meal of the day, and breakfast must provide sufficient nutrition for optimal physiological health and physical wellbeing of the body throughout the day. The effects of a healthful breakfast as observed through enhanced cognitive function would validate our hypotheses of the importance of breakfast and might prompt public health officials to begin to focus on health education regarding the value of eating a nutritious breakfast.
